# Enteric opportunistic infections in patients with inflammatory bowel disease receiving biologic therapies: a retrospective cohort study

**DOI:** 10.1186/s13099-025-00793-2

**Published:** 2025-12-21

**Authors:** Ting-Chieh Huang, Tony Kou, Chia-Jung Kuo, Cheng-Hsun Chiu, Tai-Di Chen, Chien-Ming Chen, Jen-Wei Chou, Tien-Yu Huang, Chen-Wang Chang, Cheng-Tang Chiu, Ming-Yao Su, Yu-Bin Pan, Puo-Hsien Le

**Affiliations:** 1 College of Medicine, Chang Gung University, Taoyuan City, Taiwan; 2 Department of Gastroenterology and Hepatology, Chang Gung Memorial Hospital, Linkou, Taoyuan, Taiwan; 3 Chang Gung Inflammatory Bowel Disease Center, Taoyuan, Taiwan; 4 Chang Gung Microbiota Therapy Center, Taoyuan, Taiwan; 5 Taiwan Association for the Study of Intestinal Diseases (TASID), Taoyuan, Taiwan; 6 Division of Pediatric Infectious Diseases, Department of Pediatrics, Chang Gung Memorial Hospital, Linkou, Taoyuan, Taiwan; 7 Department of Anatomic Pathology, Chang Gung Memorial Hospital, Linkou, Taoyuan, Taiwan; 8 School of Medicine, National Tsing Hua University, Hsinchu, Taiwan; 9 Department of Medical Imaging and Interventions, Chang Gung Memorial Hospital, Linkou, Taoyuan, Taiwan; 10 Center for Digestive Medicine, Department of Internal Medicine, China Medical University Hospital, Taichung, Taiwan; 11 School of Chinese Medicine, China Medical University, Taichung, Taiwan; 12 Division of Gastroenterology and Hepatology, Department of Internal Medicine, Tri-Service General Hospital, National Defense Medical Center, Taipei, Taiwan; 13 Division of Gastroenterology, Department of Internal Medicine, MacKay Memorial Hospital, Taipei, Taiwan; 14 MacKay Junior College of Medicine, Nursing and Management, Taipei, Taiwan; 15 MacKay Medical College, New Taipei City, Taiwan; 16 Division of Gastroenterology and Hepatology, Department of Internal Medicine, New Taipei Municipal Tucheng Hospital, New Taipei City, Taiwan; 17 Biostatistical Section, Clinical Trial Center, Chang Gung Memorial Hospital, Linkou, Taoyuan, Taiwan

**Keywords:** Inflammatory bowel disease, Biologics, Opportunistic infections, Cytomegalovirus, Clostridioides difficile, Clostridium innocuum

## Abstract

**Background:**

Biologic therapy has improved outcomes in inflammatory bowel disease (IBD) but may predispose patients to enteric opportunistic infections. Asian data comparing infection risk across biologic classes remain scarce. We therefore assessed the incidence of *Clostridioides difficile* infection (CDI), *Clostridium innocuum* (CI) infection, and cytomegalovirus (CMV) colitis in IBD patients treated with Vedolizumab (VDZ), anti-tumor necrosis factor agents (anti-TNF), or Ustekinumab (UST).

**Methods:**

This single‑center, retrospective cohort study included IBD patients who initiated VDZ, anti‑TNF (infliximab or adalimumab) or UST at Chang Gung IBD Center between January 2017 and December 2024. Opportunistic infection was defined as: (i) toxin‑gene PCR–positive CDI, (ii) CI isolated in stool/colonic culture, or (iii) CMV‑positive immunohistochemistry on intestinal biopsy. Incidence rates were expressed per 100 patient‑years. Infection‑free survival was compared with Kaplan–Meier analysis and log‑rank testing. Multivariable logistic regression identified independent predictors of CDI.

**Results:**

A total of 614 patients (377 Crohn’s disease; 237 ulcerative colitis) contributed 941 patient‑years of follow‑up. The incidences per 100 patient-years were 3.51 for CDI, 0.85 for CI, and 3.30 for CMV colitis. CDI and CI risks were comparable across VDZ, anti‑TNF and UST cohorts. CMV colitis was significantly more common with anti‑TNF therapy (5.9%) than with VDZ (3.4%) or UST (0.5%) (*p* = 0.020). Independent predictors of CDI were an acute IBD flare (odds ratio [OR] 3.64; 95% confidence interval 1.91–6.91), concurrent CMV colitis (OR 6.34; 95% confidence interval 2.03–19.8) and CI infection (OR 7.79; 95% confidence interval 1.40–43.3).

**Conclusion:**

VDZ and UST were not associated with excess CDI, CI or CMV risk, whereas anti‑TNF therapy conferred a higher burden of CMV colitis. Heightened infection surveillance is warranted during acute flares and refractory disease courses.

## Introduction

Biologic therapies such as anti-tumor necrosis factor (TNF) agents, anti-integrin antagonists, and anti-interleukin (IL)-12/23 antibodies have revolutionized the management of inflammatory bowel disease (IBD), leading to higher remission rates and improved patient outcomes [1–5]. However, by attenuating immune responses, these therapies may increase patients’ susceptibility to opportunistic infections. Opportunistic infections in IBD encompass a range of pathogens that exploit immunosuppression, including reactivation of latent viruses and novel intestinal bacterial pathogens. Clinical guidelines from the European Crohn’s and Colitis Organisation (ECCO) emphasize that prevention and monitoring of infections are integral to the care of IBD patients on immunomodulators or biologics [6]. Indeed, combination immunosuppressive therapy and advanced age have been identified as risk factors for opportunistic infections in IBD, with patients receiving concurrent corticosteroids, thiopurines, and biologics at particularly high risk [7]. Large cohort studies have confirmed that using multiple IBD treatments (e.g. an anti-TNF plus an immunomodulator) confers a higher infection risk than monotherapy [8]. In contrast, newer gut-selective agents like vedolizumab may carry a more favorable infection profile, although real-world data are still emerging [9, 10]. Overall, the balance of benefit and infection risk must be carefully considered with biologic therapy in IBD [8].

Among opportunistic infections, gastrointestinal pathogens are of special concern in IBD patients on biologics. *Clostridioides difficile* infection (CDI) is a well-recognized complication that can precipitate IBD flares and worsen disease outcomes. IBD patients have a significantly higher incidence of CDI compared to the general population – on the order of a 2- to 5-fold increased risk in some studies [11, 12]. Notably, immunosuppressive therapy appears to amplify this risk. In a population-based cohort study from Manitoba, systemic corticosteroid therapy and exposure to anti-TNF biologics - specifically infliximab (IFX) or adalimumab (ADA) - were each independent predictors of CDI in patients with IBD [12]. In patients with IBD, CDI is associated with a more aggressive clinical course, characterized by earlier onset during hospitalization, increased need for intensive therapy, and significantly higher mortality rates than those observed in non-IBD populations [11–13]. These observations underscore the importance of vigilant monitoring for CDI in patients receiving biologics, especially anti-TNFs. In contrast, the risk of CDI with the gut-selective agent vedolizumab (VDZ) remains controversial. While several meta-analyses and retrospective cohort studies suggest that VDZ does not significantly increase CDI risk compared to anti‑TNF agents or 5-aminosalicylic acid (5-ASA) therapy, other investigations have reported a possible increased incidence, indicating heterogeneity among study outcomes. For example, a 2024 systematic review and meta-analysis in ulcerative colitis found no significant association between VDZ and CDI risk [14]. Conversely, a subset of cohort studies has signaled a higher CDI rate with VDZ relative to anti-TNF therapy in ulcerative colitis (UC) [15]. Notably, a recent meta-analysis including 41,862 patients reported an overall CDI incidence of 1.3% under vedolizumab treatment, with UC patients exhibiting a more than two‑fold higher risk compared to Crohn’s disease (CD) (relative risk [RR] 2.25; 95% confidence interval 1.73–2.92) [16]. Thus, conflicting outcomes warrant further prospective investigation. By contrast, data on ustekinumab (UST) and CDI incidence are limited. Available safety analyses, including pooled trial data, report a low incidence of CDI events in UST-treated IBD patients, yet no dedicated studies have formally assessed its impact [17]. Consequently, the role of UST in CDI risk remains to be elucidated in future research.

In recent years, *Clostridium innocuum* (CI) has emerged as an opportunistic enteric pathogen in IBD [18]. CI is an anaerobic, vancomycin-resistant bacterium that has been implicated in refractory IBD [18]. Studies have found that in UC, CI infection is associated with reduced likelihood of achieving clinical remission, and in CD it correlates with complications such as creeping fat and stricture formation [19]. However, the overall incidence of CI infection in IBD on biologics and its relative frequency under different therapies remain poorly characterized.

Another key opportunistic pathogen in IBD is cytomegalovirus (CMV). Severe UC—particularly when patients are exposed to high-dose corticosteroids or biologic agents—facilitates reactivation of latent CMV in colonic tissue [6]. CMV colitis can masquerade as, or amplify, an IBD flare and frequently prompts the addition of antiviral therapy to standard immunosuppression [20]. Its presence is consistently linked to worse clinical outcomes, including reduced response to medical rescue, longer hospital stays and a higher likelihood of IBD-related surgery [21]. Notably, a Japanese pathology-based series detected CMV in 21% of IBD related surgery specimens obtained from steroid-refractory UC, underscoring its clinical relevance [22]. As the use of biologics grows, systematic CMV screening has become standard in acute severe or treatment-refractory colitis; nevertheless, head-to-head real-world comparisons of CMV incidence across different biologic classes remain sparse. Overall, biologic-treated IBD patients remain vulnerable to a spectrum of opportunistic infections—most prominently *C. difficile*, CI, and CMV—whose impact on gastrointestinal outcomes mandates vigilant surveillance. Although traditional infection risk factors in IBD are well documented, direct comparisons of infection rates among anti-TNF, anti-integrin, and anti-IL-12/23 agents are limited, and predictors of specific infections such as CDI in the era of advanced biologics remain incompletely defined. To address these gaps, we conducted a retrospective cohort study to quantify opportunistic infection incidence in biologic-treated IBD and to determine whether risk varies by biologic class.

## Materials and methods

### Study design and patients

This retrospective cohort study included adults with a confirmed diagnosis of IBD who initiated biologic therapy at Linkou Chang Gung Memorial Hospital between October 2017 and November 2024. UC and CD were diagnosed according to standard clinical, endoscopic and histopathological criteria. Eligible patients received at least one of the following biologic agents: VDZ, IFX, ADA or UST. Follow-up extended from biologic initiation until drug discontinuation, loss to follow-up, or 30 November 2024, whichever occurred first. Exclusion criteria were incomplete medical records, pregnancy and participation in clinical trials of investigational drugs.

## Data collection and outcome

Demographic data (age, sex, body-mass index), disease characteristics (IBD subtype and duration), and treatment details (specific biologic, cumulative exposure, and concomitant corticosteroid, immunomodulator, or antibiotic use) were extracted from electronic medical records. The primary outcomes were incident CDI, CI infection, and CMV infection. Secondary outcomes included steroid-free clinical remission at week 52, physician-documented acute flares, IBD-related hospitalization, and complications such as stricture, perforation, abscess, fistula, or IBD-related surgeries.

## Definitions

CDI was defined as diarrhea with a positive stool toxin-gene PCR for *C. difficile*. CMV infection required characteristic cytopathic changes or positive immunohistochemistry for CMV antigen on colonic biopsy. CI infection was confirmed by isolation of the organism from stool culture in symptomatic patients. The same diagnostic criteria for CDI, CMV colitis, and CI infection were uniformly applied across all biologic treatment groups, in accordance with international guidelines [6, 23, 24]. Steroid-free clinical remission was defined as achieving clinical remission at week 52 after biologic initiation without the use of systemic corticosteroids. This definition reflects a single time-point assessment, rather than indicating durable steroid-free remission across multiple preceding intervals. Such a time-point–based approach is consistent with the methodological framework commonly adopted in major IBD clinical trials and endorsed by international guideline bodies, including ECCO and American Gastroenterological Association (AGA) [3–26]. An IBD flare-up was defined as new or worsening gastrointestinal symptoms (e.g., increased stool frequency, rectal bleeding, abdominal pain, or urgency) after a period of clinical stability, together with at least one objective marker of intestinal inflammation such as elevated C-reactive protein (CRP), increased fecal calprotectin (FCP), or endoscopic or radiologic evidence of active disease. Primary nonresponder was defined as the absence of clinical response at the end of induction (week 14 for anti-TNF agents and vedolizumab, and week 16 for ustekinumab), together with objective evidence of active inflammation or treatment discontinuation due to lack of efficacy. Secondary nonresponder was defined as an initial clinical improvement followed by symptomatic relapse with objective markers of inflammation during maintenance, requiring dose escalation, switching, or treatment discontinuation.

### Statistical analysis

Infection incidence was expressed as events per 100 patient-years (PY) for each biologic. Incidence rate ratios were compared under a Poisson distribution using the χ² test, and infection-free survival was evaluated with Kaplan–Meier curves and the log-rank test. Variables associated with CDI at *p* < 0.10 in univariate analysis were entered into a multivariable logistic-regression model; a two-sided *p* < 0.05 was deemed statistically significant. All analyses were conducted with SPSS version 30.0 (IBM Corp., Armonk, NY, USA).

## Results

### Baseline characteristics

A total of 614 biologic-treated IBD patients were analyzed—306 with UC and 308 with CD. Exposure comprised 238 VDZ, 186 anti-TNF (IFX or ADA) and 190 UST courses. VDZ was prescribed more frequently for UC (60.9%, *n* = 145), whereas anti-TNF and UST predominated in CD (66.7%, *n* = 124, and 84.2%, *n* = 160, respectively; *p* < 0.001). VDZ recipients were older (47.9 ± 15.1 year) than anti-TNF (42.8 ± 16.8 year) or UST users (43.6 ± 16.5 year; *p* < 0.001). Sex distribution (male 69.1%), body mass index (BMI) (22.9 ± 4.2) and baseline systemic-steroid exposure did not differ among groups. Ever-smoker status varied among biologic groups (overall 11.6%, *n* = 71, *p* = 0.004), with the highest prevalence in UST users (17.4%, *n* = 33), followed by VDZ (10.9%, *n* = 26) and anti-TNF (6.5%, *n* = 12) (Table 1). Among patients who developed enteric opportunistic infections, three had received systemic antibiotics within the 30 days prior to the index infection. In the overall cohort, antibiotic exposure during biologic therapy was rare (3/614, < 1%), precluding meaningful between-group comparisons or its inclusion as an independent variable in multivariable analysis.

**Table 1:** Baseline Demographics, concomitant Medications, and 52-Week outcomes stratified by biologic class

	Overall (*n* = 614)	Vedolizumab (*n* = 238)	Anti TNF (*n* = 186)	Ustekinumab (*n* = 190)	*p* value
**Baseline characteristics**					
Age (mean ± SD years)	45.01 ± 16.20	47.87 ± 15.130	42.77 ± 16.807	43.61 ± 16.459	< 0.001*
Gender (male)	424 (69.1%)	157 (66%)	130 (69.9%)	137(72.1%)	0.426
Body mass index (mean ± SD, kg/m^2^)	20.33 ± 8.41	22.79 ± 4.14	22.38 ± 4.17	22.03 ± 3.96	0.125
Crohn’s disease	377(61.4%)	93(39.1%)	124 (66.7%)	160(84.2%)	< 0.001*
Ever-Smoker	71(11.6%)	26(10.9%)	12(6.5%)	33(17.4%)	0.004*
Other medications					
5-ASA	412 (67.2%)	174 (73.4%)	127 (68.3%)	111(58.4%)	< 0.001*
Steroid	393 (64.0%)	166 (69.7%)	113(60.8%)	114(60%)	0.321
Immunosuppressants	185(30.1%)	61(25.6%)	71(38.2%)	53(27.9%)	0.004*
Antibiotics	3(0.5%)	1(0.4%)	0	2(1%)	0.336
**Outcomes (52 weeks)**					
Persistence	481 (78.3%)	177 (74.4%)	132(71%)	172(90.5%)	< 0.001*
Reasons of Discontinuation					
Side effect	10(1.6%)	2(0.84%)	7(3.76%)	1(0.53%)	0.0218*
Primary nonresponder	17(2.77%)	13(5.46%)	2(1.08%)	2(1.05%)	0.0053*
Secondary nonresponder	85(13.84%)	38(15.97%)	37(19.89%)	10(5.26%)	< 0.001*
Personal Reasons	5(0.81%)	3(1.26%)	2(1.08%)	0	0.316
Others	16(2.6%)	5(2.1%)	6(3.23%)	5(2.63%)	0.771
**Steroid-free clinical remission**	416 (67.8%)	170 (71.4%)	128(68.8%)	118(62.1%)	0.190
IBD related admission	125(20.4%)	52(21.8%)	44(23.7%)	29(15.3%)	0.109
Etiologies					
Opportunistic infections	62 (10.2%)	23 (9.7%)	24 (13.1%)	15(7.9%)	0.380
IBD related complications ^a^	10 (1.6%)	3 (1.3%)	4 (2.2%)	3(1.6%)	0.343
IBD related surgeries	25(4.1%)	14(5.9%)	10(5.4%)	1(0.5%)	0.006*
Acute flare up	76(12.4%)	32(13.45%)	21(11.29%)	23(12.1%)	0.897

a. IBD related complications included fistula, lumen stricture, intra-abdominal abscess, free perforation, and toxic megacolon. Antibiotic use was defined as systemic antibiotic exposure within 30 days preceding the index enteric opportunistic infection

IBD: Inflammatory Bowel Disease; SD: standard deviation༛TNF: tumor necrosis factor༛5-ASA: 5-Aminosalicylic Acid

## Incidence of opportunistic infections

During 941 patient-years of follow-up (Table 2), the incidence of CDI was 4.13 events/100 PY in VDZ users, 2.72 events/100 PY in anti-TNF users and 3.17 events/100 PY in UST users, with no significant inter-group difference (*p* = 0.602). CI infection remained rare, occurring at 1.10, 0.68 and 0.70 events/100 PY, respectively (*p* = 0.802). In contrast, CMV infection occurred significantly more often among anti-TNF users (3.74 events/100 PY) than among those receiving VDZ (2.20 events/100 PY) or UST (0.35 events/100 PY; *p* = 0.020).

**Table 2:** Incidence of opportunistic infections (events / 100 patient-years) in IBD patients stratified by biologic agents

Incidence	Overall (PY = 941)	Vedolizumab (PY = 363)	Anti TNF (PY = 294)	Ustekinumab (PY = 284)	*p* value
Incidence					
*Clostridioides difficile*	33 (3.51%)	15 (4.13%)	8 (2.72%)	9 (3.17%)	0.602
*Clostridium innocuum*	8 (0.85%)	4 (1.1%)	2 (0.68%)	2 (0.7%)	0.802
Cytomegalovirus	20 (3.3%)	8 (3.4%)	11 (5.9%)	1 (0.5%)	0.020*

IBD: Inflammatory Bowel Disease; TNF: tumor necrosis factor༛PY: Patient-years

## Time-to-Infection analysis

**Fig. 1:**
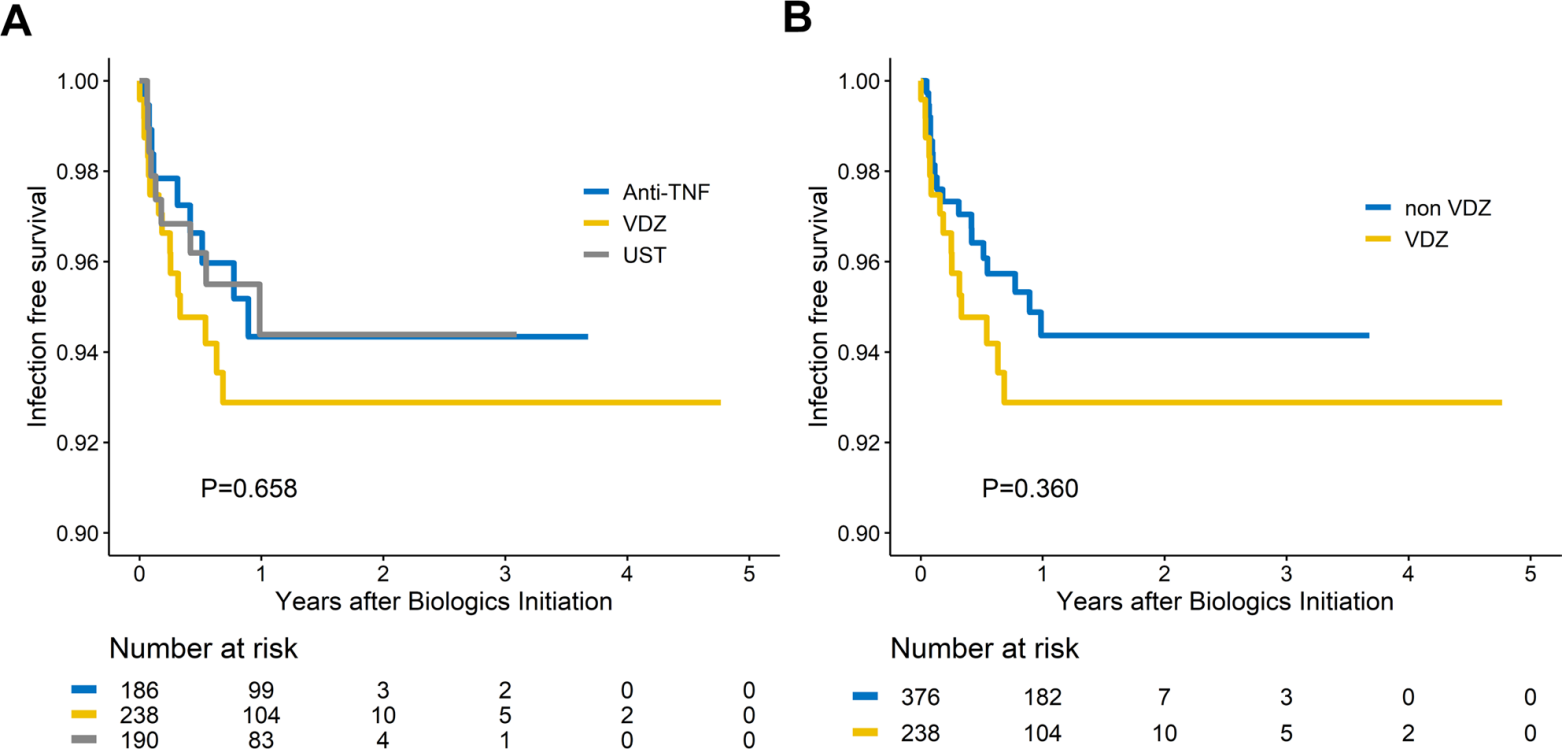
Time to *Clostridioides difficile* infection. (**A**) Kaplan–Meier curves stratified by biologic class ( vedolizumab[VDZ], anti-tumor necrosis factor[TNF], ustekinumab[UST]) show no significant difference in CDI-free survival. (**B**) Comparison of VDZ versus non-VDZ exposure likewise reveals no significant difference in time to CDI

**Fig. 2:**
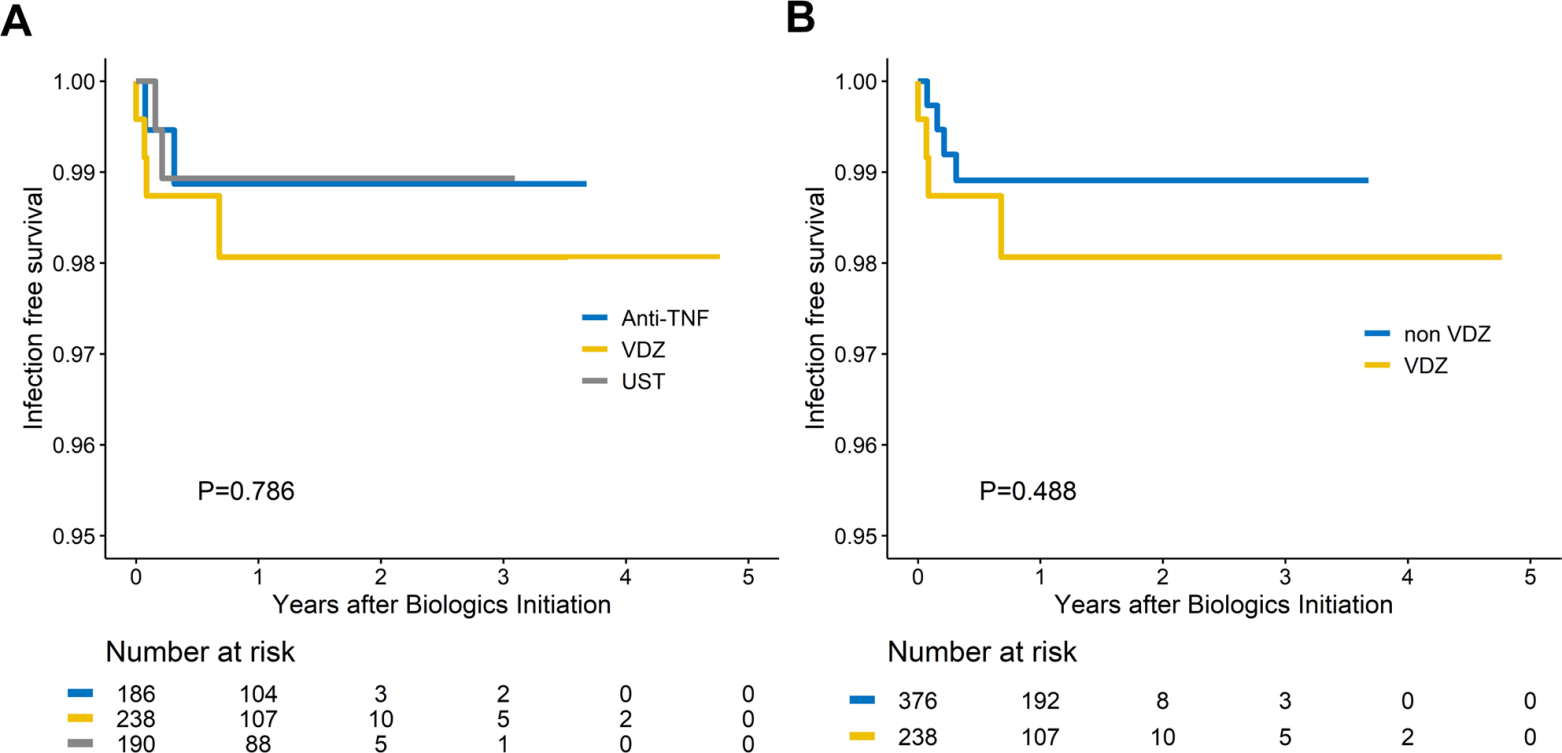
Time to *Clostridium innocuum* infection. (**A**) Kaplan–Meier analysis demonstrates equivalent CI-free survival across vedolizumab (VDZ), anti-tumor necrosis factor (TNF), and ustekinumab (UST) groups. (**B**) CI-free survival does not differ between VDZ and non-VDZ users

**Fig. 3:**
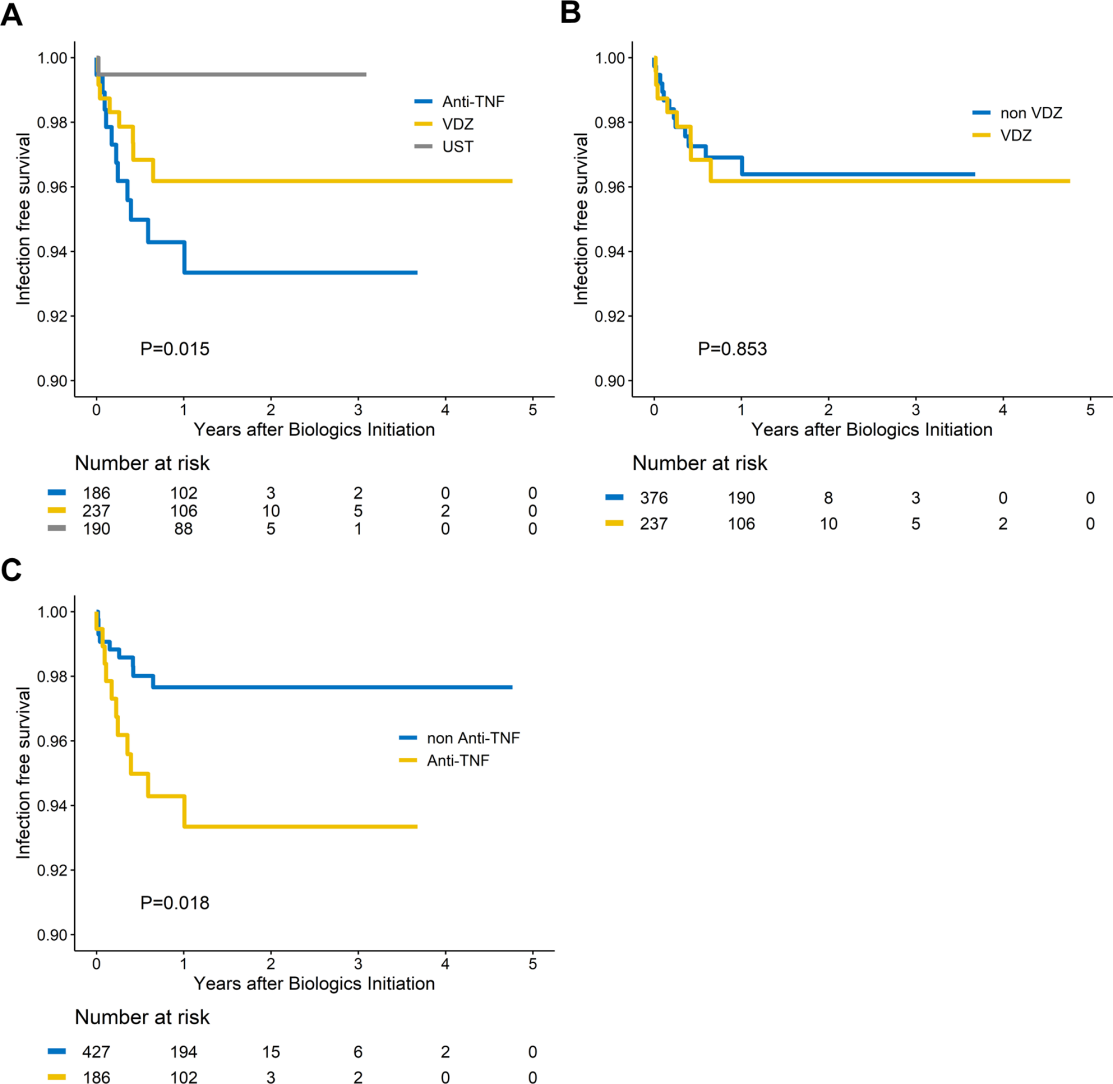
Time to cytomegalovirus (CMV) infection. (**A**) Anti- tumor necrosis factor (TNF) therapy is associated with significantly earlier CMV infection compared with vedolizumab (VDZ) or ustekinumab (UST) (log-rank *p* < 0.05). (**B**) No difference in CMV-free survival is observed between VDZ and non-VDZ users. (**C**) Anti-TNF users exhibit earlier CMV infection than patients receiving non-anti-TNF biologics (log-rank *p* < 0.05) Abbreviations: IBD: inflammatory bowel disease; TNF : tumor necrosis factor; UST : ustekinumab; VDZ : vedolizumab; CMV: cytomegalovirus

Kaplan–Meier curves (Figs. 1A, 2 and 3C) demonstrated no difference in CDI-free survival among the three biologic classes (log-rank *p* = 0.658) or between VDZ and non-VDZ exposure (log-rank *p* = 0.360). CI-free survival was likewise comparable (log-rank *p* = 0.965 across agents; *p* = 0.791 for VDZ vs. non-VDZ). CMV infection occurred earlier with anti-TNF therapy than with VDZ or UST (log-rank *p* = 0.015) and remained more frequent when anti-TNF was compared with non-anti-TNF regimens (log-rank *p* = 0.018); no difference emerged between VDZ and non-VDZ groups (log-rank *p* = 0.853).

### Predictors of CDI

On univariate analysis, CDI was associated with acute flare (odds ratio [OR] 5.344, *p* < 0.001), CMV co-infection (OR 9.346, *p* < 0.001), CI co-infection (OR 11.520, *p* < 0.001) and absence of steroid-free remission at week 52 (OR 0.374, *p* = 0.006), whereas concomitant immunomodulator use was protective (OR 0.305, *p* = 0.028). Multivariable logistic regression (Table 3) confirmed acute flare (OR 3.639, 95% confidence interval 1.569–8.444, *p* = 0.003), CMV infection (OR 6.339, 95% confidence interval 2.052–19.582, *p* = 0.001) and CI infection (OR 7.785, 95% confidence interval 1.231–49.221, *p* = 0.029) as independent predictors of CDI. Biologic class, including VDZ (OR 1.338, *p* = 0.419), did not independently influence CDI risk.

**Table 3:** Logistic regression analysis of risk factors for *Clostridioides difficile* infection in Biologic-Treated IBD patients

	Univariate analysis			Multivariate analysis		
	OR	95% CI	*p* value	OR	95% CI	*p* value
Gender (male)	1.425	0.631–3.221	0.394			
BMI	0.994	0.954–1.036	0.774			
Crohn’s disease	0.652	0.323–1.317	0.233			
Ever-smoker	0.479	0.112–2.045	0.320			
Age	1.016	0.995–1.038	0.130			
Biologic agents						
Anti-TNF	0.856	0.390–1.879	0.698			
Vedolizumab	1.338	0.661–2.708	0.419			
Ustekinumab	0.829	0.378–1.819	0.639			
Other IBD medication						
5-ASA	1.867	0.796–4.377	0.151			
Steroid	1.192	0.818–1.737	0.361			
Immunosuppressants	0.305	0.106–0.880	0.028*	0.351	0.117–1.054	0.062
Steroid free clinical remission	0.374	0.184–0.759	0.006*	0.677	0.303–1.513	0.342
Acute flare up	5.344	2.536–11.265	< 0.001*	3.639	1.569–8.444	0.003*
IBD related complication^b^	1.986	0.244–16.161	0.521			
CMV infection	9.346	3.323–26.284	< 0.001*	6.339	2.052–19.582	0.001*
*C. innocuum* infection	11.52	2.629–50.489	< 0.001*	7.785	1.231–49.221	0.029*

b. IBD related complications included fistula, lumen stricture, intra-abdominal abscess, free perforation, and toxic megacolon. Antibiotic use was defined as systemic antibiotic exposure within 30 days preceding the index enteric opportunistic infection

IBD: Inflammatory Bowel Disease; SD: standard deviation༛TNF: tumor necrosis factor༛5-ASA: 5-Aminosalicylic Acid༛CMV: Cytomegalovirus༛*C. innocuum* : *Clostridium innocuum*༛CI: confidence interval

### Secondary outcomes

In addition to infection incidence, several secondary outcomes were evaluated to provide a broader perspective on treatment response and infection risk. Treatment persistence, IBD-related hospitalization, IBD-related surgery, and steroid-free clinical remission at week 52 were assessed across biologic groups (Table 1). UST demonstrated the highest treatment persistence (90.5%) and the lowest rate of IBD-related surgery (0.5%), suggesting more stable disease control compared with VDZ (74.4%) and anti-TNF (71%) users. Rates of acute flare and IBD-related admission were similar among groups. Steroid-free clinical remission was achieved in 67.8% of the overall cohort, and its absence was significantly associated with CDI in univariate analysis, although not retained in the multivariable model.

## Discussion

In this retrospective cohort of biologic-treated IBD patients, vedolizumab was not associated with an increased risk of CDI, CI, or CMV infection. This finding concurs with numerous global studies that highlight VDZ’s favorable infection safety profile, often attributed to its gut-selective mechanism that spares systemic immunity. Clinical trials and real-world cohorts (e.g., the VICTORY consortium) have reported low serious infection rates with VDZ [27], and a recent meta-analysis found no significant elevation in CDI risk among vedolizumab-treated patients [16]. Our results reinforce these trends, suggesting that VDZ’s intestinally targeted action can effectively control inflammation without substantially predisposing to opportunistic infections. This is especially reassuring in an Asian population, providing evidence that VDZ’s safety extends to a region where real-world data have been limited.

It should be noted, however, that not all studies are uniform in this regard. For instance, a Spanish GETECCU registry cohort (CDIFVEDO) raised concern for a higher incidence of CDI in VDZ users with moderate-to-severe UC^15^. Moreover, an analysis from the Swedish national register (SWIBREG) reported an unexpected finding: in CD, VDZ-treated patients had a higher hazard of serious infections (notably gastrointestinal infections) compared to those on anti-TNFs [28]. No such difference was observed in UC in that study. These discrepancies underscore that infection outcomes can vary by population and disease phenotype. Differences in baseline disease severity, concomitant medications, and regional pathogen exposure—particularly variations in gut microbiota or the prevalence of toxigenic C. difficile strains—may account for the divergent results. Notably, our Asian cohort did not detect an increased infection risk with VDZ, suggesting that in real-world Asian practice VDZ remains as infection-safe as other biologics, despite the higher CDI signals reported in some Western studies.

In contrast to VDZ, anti-TNF therapy was linked to a higher burden of CMV colitis in our study. CMV infection occurred in about 5.9% of anti-TNF–treated patients, significantly more frequent than in those on VDZ (3.4%) or UST (0.5%). This trend aligns with the well-documented role of TNF-α in antiviral immune defense: TNF-α blockade can impair cytotoxic T-cell responses and allow viral reactivation [6]. Indeed, anti-TNF agents have previously been associated with opportunistic viral infections, particularly when used concomitantly with other immunosuppressants or in the setting of severe disease activity [6]. Consistent with the 2021 ECCO infection guideline, which advocates systematic CMV testing in episodes of acute severe UC before or during escalation to biologics, our findings underscore the importance of such screening—particularly in patients receiving anti-TNF therapy [6]. The excess CMV burden observed in anti-TNF users underscores the need for stringent viral surveillance—such as periodic CMV testing throughout anti-TNF treatment—and for initiating antiviral therapy promptly to avert CMV-related complications and safeguard IBD outcomes [29]. UST, an anti-IL12/23 antibody, demonstrated a similarly low opportunistic infection incidence as VDZ in our cohort. We observed no excess CDI, CI, or CMV risk with UST – notably, CMV colitis was rarest in the UST group. This aligns with pooled global safety analyses showing UST has a low rate of serious infections in both CD and UC, even up to 4–5 years of use [17]. A large Korean nationwide study likewise found non-anti-TNF biologics (VDZ/UST) tended to be associated with a lower risk of serious infection or active tuberculosis than anti-TNF-α agents [30]. Together, these findings suggest that newer biologic classes (anti-integrin and anti-IL12/23) may confer a safety advantage over anti-TNFs regarding systemic and opportunistic infections – a consideration that could inform biologic selection, particularly in regions like Asia where infections such as tuberculosis remain a concern.

In our series, CDI occurred at ~ 3–4 events per 100 PY, a frequency well within the range reported for biologic-treated IBD. By comparison, a recent global meta-analysis estimated that 8–11% of IBD patients experience at least one episode of CDI over the course of disease, with comparable proportions across Europe, North America and Asia—underscoring the worldwide relevance of CDI surveillance in IBD management [31]. Crucially, we found no significant difference in CDI rates across biologic therapies (anti-TNF, VDZ, or UST), indicating that the choice of biologic class did not markedly influence CDI risk in our setting. Beyond comparing infection rates by therapy, our study yields important insights into risk factors for opportunistic infections in IBD. In multivariable analysis, acute IBD flare-ups emerged as a strong independent predictor of CDI (OR 3.6).

This relationship is well-supported by literature: an IBD flare can disrupt the mucosal barrier and alter the gut microbiome, often necessitating corticosteroids or antibiotics – all of which create a permissive environment for *C. difficile* colonization and toxin production [32]. Reflecting this pathophysiology, the 2021 American College of Gastroenterology (ACG) guideline on CDI recommends that every patient with new-onset or worsening IBD symptoms be tested for CDI, because prompt detection and appropriate antimicrobial therapy are associated with improved clinical outcomes [23]. Clinically, flare-ups may also mask or mimic CDI symptoms, leading to delayed diagnosis and management. Our findings emphasize that an acute exacerbation in IBD should raise immediate suspicion for superimposed CDI. Early stool testing and preemptive infection control (e.g. isolation and empiric therapy) during severe flares could improve outcomes, given that undiagnosed CDI in IBD is associated with worse clinical courses, including higher IBD-related surgeries and mortality rates [33, 34]. Fecal microbiota transplantation (FMT) should be considered in patients with IBD with refractory or recurrent CDI to improve clinical outcomes [35]. We also found that concurrent viral infection with CMV significantly predisposed patients to CDI (OR 6.3). Although CDI and CMV colitis are each known complications in IBD, their coexistence creates a particularly challenging scenario. CMV infection can exacerbate colonic inflammation and complicate the clinical picture, making it harder to recognize an underlying *C. difficile* infection. Reports have linked combined CMV–CDI in colitis to heightened morbidity, such as increased need for intensive medical therapy or surgery [36, 37].

It is plausible that severe colitis driven by CMV provides a nidus for *C. difficile* overgrowth (through mucosal damage and broad antibiotic use), and conversely, CDI’s toxin-mediated inflammation might promote viral reactivation. Our results underscore the importance of dual surveillance: in refractory IBD flares, especially under biologic or steroid treatment, clinicians should test for both CMV and *C. difficile*. Early detection of coinfections enables timely antiviral and antibiotic therapy, which may prevent further deterioration. Perhaps most novels are the identification of CI infection as an independent predictor of CDI. Patients in our cohort who grew CI had markedly higher odds of concurrent *C. difficile* infection (OR 7.8). CI is an emerging anaerobe in IBD, recognized as a cause of antibiotic-associated diarrhea and refractory colitis.

Unlike *C. difficile*, CI produces no toxins, yet it has been associated with severe intestinal disease and even systemic infections [38–41]. Notably, CI is intrinsically resistant to vancomycin, meaning standard CDI therapy can fail if CI is the true culprit. Our findings suggest that CI and *C. difficile* infections often overlap in high-risk IBD patients, possibly reflecting a shared predisposition (e.g. broad antibiotic exposure or disrupted gut flora). Intriguingly, a recent study from our center reported that in hospitalized IBD patients, CI infections were detected even more frequently than *C. difficile*, and were associated with greater disease severity [18]. Beyond simple coexistence, emerging mechanistic data support a deeper biological link between CI, dysbiosis, and intestinal barrier disruption. One recent study demonstrated that viable gut bacteria can translocate from the inflamed intestine to mesenteric adipose tissue and drive the formation of creeping fat, providing direct evidence that dysbiosis-associated organisms can disseminate beyond the gut lumen [19]. Such translocation could facilitate mixed polymicrobial infections in severe CD and create a permissive niche for CDI, offering a plausible explanation for the high CI–CDI co-infection rates observed across clinical cohorts. These observations underscore that CI is a bona fide pathogen in IBD rather than an innocuous bystander.

For practitioners, the co-occurrence of these infections means that an IBD patient not responding to conventional CDI therapy might warrant stool culture for CI or molecular testing. Conversely, when CI is identified, one should also suspect coexistent CDI, given their symptomatic overlap. In sum, recognizing CI as part of the “opportunistic infection spectrum” in IBD is important for tailoring antibiotic therapy (e.g. considering fidaxomicin or fecal microbiota transplantation in vancomycin-resistant cases).

Finally, in our cohort, CI and CDI were typically identified from the same index stool specimen or within a narrow diagnostic interval, without any reproducible temporal pattern indicating that one infection consistently preceded the other. This supports the interpretation that CI and CDI most often arise concurrently in the context of shared underlying risk factors, rather than representing a sequential infectious process. Because our primary outcome was the overall incidence of enteric opportunistic infections (CDI, CI, and CMV colitis), potential confounding by antibiotic exposure warrants consideration. Antibiotics are a well-established risk factor for CDI in IBD [6, 23], whereas their role in non-CDI enteric infections such as CI or CMV colitis is less certain. In our cohort, antibiotic use was rare- only three infected patients had antecedent (≤ 30-day) exposure, and overall on-treatment exposure was < 1% - which likely limited the ability to detect any meaningful association. It is also possible that some antibiotic prescriptions were issued outside our institution or more than one month before infection onset, which could not be captured in this dataset. These factors likely contributed to the lack of observable associations between antibiotic exposure and infection risk in this cohort. Such limitations may have diluted or distorted the associations between biologic class and infection risk despite adjustment strategies, thereby warranting cautious interpretation of the effect estimates [7–9, 28, 42].

Beyond infection incidence, several secondary outcomes provide additional perspective on treatment effectiveness and infection susceptibility. UST showed higher treatment persistence and lower IBD-related surgery rates, suggesting more stable disease control and potentially fewer infection-related complications. Rates of flare and hospitalization were comparable across biologic groups, indicating that inflammatory activity—rather than biologic class itself—appears to be the primary driver of infection risk. Moreover, steroid-free clinical remission, achieved by approximately two-thirds of patients, represents a favorable treatment endpoint reflecting both adequate disease control and reduced vulnerability to opportunistic infections through avoidance of corticosteroid-induced immunosuppression. Collectively, these findings complement the primary infection analysis and emphasize the interplay between disease control, immunosuppression, and infection risk in biologic-treated IBD. Overall, our study provides a valuable regional perspective on biologic safety and opportunistic infections. Asian IBD patients have been underrepresented in many registries and trials; hence, our data offer novel insights for a population with rising IBD prevalence. We demonstrated that infection patterns in Asian patients on biologics largely mirror those reported globally - VDZ and UST appear infection-safe, while anti-TNF therapy warrants caution for viral infections – but we also highlighted nuances like the prominent role of CI. These findings carry practical implications. First, they support the use of gut-selective or IL-12/23 agents in patients where infection risk is a concern (for example, older patients or those with a history of opportunistic infections), without compromising infection-free outcomes. Second, they stress the need for rigorous infection surveillance and prophylaxis during acute flares and refractory disease courses, regardless of biologic class. Given the expanding armamentarium of IBD therapies and the increasing incidence of IBD in Asia, our results contribute to evidence-based decision-making for personalized, infection-conscious care in the biologic era.

This study has several limitations. First, it is a retrospective, single-center analysis conducted in a tertiary IBD referral hospital, which may limit generalizability, as such centers often care for patients with more severe, refractory, or clinically complex disease.Second, the proportions of UC and CD differed across biologic groups, and detailed histories of prior biologic exposure were unavailable. Because our analysis focused on infection risk at the time of index biologic treatment, historical biologic exposure was not systematically collected. Third, although CI and CMV were independent predictors of infection risk, their absolute event numbers were small, limiting power for subgroup analyses. Diagnoses of opportunistic infections relied on routine clinical and microbiological documentation; variability in clinician testing thresholds and diagnostic methods raises the possibility of ascertainment bias. Fourth, antibiotic exposure—a well-established risk factor for CDI in IBD [6, 23]—was incompletely captured. Antibiotic use was rare in this cohort, reducing power to detect meaningful associations. Some prescriptions may have occurred outside our institution or beyond the one-month capture window before infection onset and therefore were unrecorded. Detailed data on class, dose, indication, and timing relative to biologic initiation were also unavailable. These limitations may have attenuated detectable associations and are shared by many high-impact retrospective and registry-based studies [7–9, 28, 42]. More complete and temporally granular antibiotic documentation would enhance future analyses [43]. Finally, this study assessed only three pathogens (CDI, CMV, and CI); fungal, mycobacterial, and other viral infections were not evaluated. Although age and BMI were and showed no association with infection risk, additional host factors—such as frailty, perianal disease, or very low BMI—were not systematically recorded and therefore could not be incorporated into multivariable models. Broader pathogen surveillance and more comprehensive host- and treatment-related data will be necessary to strengthen comparability and causal inference in future research. 

## Conclusions

In this Asian cohort, VDZ and UST were not linked to excess CDI, CI or CMV, whereas anti-TNF therapy carried a higher CMV burden. Acute flares, CMV and CI emerged as independent predictors of CDI, underscoring the need for vigilant dual-pathogen screening during severe disease activity. Prospective, multicenter studies that include a broader range of opportunistic infections—as well as detailed antibiotic and immunomodulator data—are warranted to confirm these findings and refine infection-focused biologic selection strategies.

## Data Availability

The datasets used and/or analysed in the current study are available from the corresponding author upon reasonable request.

## Abbreviations

IBD: Inflammatory bowel disease
CDI: Clostridioides difficile infection
CI: Clostridium innocuum
CMV: Cytomegalovirus
VDZ: Vedolizumab
TNF: Tumor necrosis factor
UST: Ustekinumab
OR: Odds ratio
IL: Interleukin
ECCO: European Crohn’s and Colitis Organisation
IFX: Infliximab
ADA: Adalimumab
5-ASA: 5-Aminosalicylic acid
UC: Ulcerative colitis
CD: Crohn’s disease
RR: Relative risk
PY: Patient-years
BMI: Body mass index
ACG: American College of Gastroenterology
FMT: Fecal microbiota transplantation
